# Sleep Duration and Insomnia in Adolescents Seeking Treatment for Anxiety in Primary Health Care

**DOI:** 10.3389/fpsyg.2021.638879

**Published:** 2021-03-24

**Authors:** Bente S. M. Haugland, Mari Hysing, Valborg Baste, Gro Janne Wergeland, Ronald M. Rapee, Asle Hoffart, Åshild T. Haaland, Jon Fauskanger Bjaastad

**Affiliations:** ^1^Department of Clinical Psychology, Faculty of Psychology, University of Bergen, Bergen, Norway; ^2^Regional Centre for Child and Youth Mental Health and Child Welfare, Norwegian Research Centre, Bergen, Norway; ^3^Department of Psychosocial Science, Faculty of Psychology, University of Bergen, Bergen, Norway; ^4^Department of Child and Adolescent Psychiatry, Division of Psychiatry, Haukeland University Hospital, Bergen, Norway; ^5^Department of Clinical Medicine, Faculty of Medicine, University of Bergen, Bergen, Norway; ^6^Centre for Emotional Health, Department of Psychology, Macquarie University, Sydney, NSW, Australia; ^7^Research Institute, Modum Bad Psychiatric Hospital, Vikersund, Norway; ^8^Department of Psychology, University of Oslo, Oslo, Norway; ^9^Sorlandet Hospital HF, Kristiansand, Norway; ^10^Division of Psychiatry, Stavanger University Hospital, Stavanger, Norway

**Keywords:** adolescents, anxiety symptoms, depressive symptoms, primary health care, insomnia, sleep onset latency, sleep duration

## Abstract

There is limited knowledge about sleep in adolescents with elevated levels of anxiety treated within primary health care settings, potentially resulting in sleep problems not being sufficiently addressed by primary health care workers. In the current study self-reported anxiety, insomnia, sleep onset latency, sleep duration, and depressive symptoms were assessed in 313 adolescents (12–16 years; mean age 14.0, *SD* = 0.84, 84.0% girls) referred to treatment for anxiety within primary health care. Results showed that 38.1% of the adolescents met criteria for insomnia, 34.8% reported short sleep duration (<7 h), and 83.1% reported long sleep onset latency (≥30 min). Total anxiety symptoms were related to all sleep variables after controlling for age and sex. Furthermore, all anxiety symptom sub-types were associated with insomnia and sleep onset latency, whereas most anxiety subtypes were associated with sleep duration. Adolescents’ depressive symptoms accounted for most of the anxiety-sleep associations, emphasizing the importance of depressive symptoms for sleep. However, anxiety was associated with insomnia and sleep onset latency also among youth with low levels of depressive symptoms. The findings suggests that primary health care workers should assess sleep duration, sleep onset latency, and insomnia in help-seeking adolescents with anxiety.

## Introduction

Sleep undergoes major changes during adolescence, and is generally characterized by short sleep duration, long sleep onset latency (SOL), and high rates of insomnia (Gradisar et al., 2011; Hysing et al., 2013). Research indicates that youth with anxiety disorders have even higher rates of insomnia (Johnson et al., 2006a; Alvaro et al., 2017), longer SOL (Forbes et al., 2008) and shorter sleep duration compared to their non-anxious peers (Roberts and Duong, 2017; Zhang et al., 2017).

Clinical research on the association between anxiety and sleep problems has primarily focused on adolescents with anxiety disorders, often in specialized treatment settings (Brown et al., 2018). We know much less about sleep in adolescents with anxiety receiving treatment within primary health care services. Such information is important because a large proportion of adolescents with symptoms of internalizing disorders (i.e., anxiety disorders and/or depression) seek help within primary health settings (Zachrisson et al., 2006). Furthermore, primary health care interventions may reach adolescents with anxiety who may not otherwise receive help (Husabo et al., 2020). By reaching adolescents early and within their everyday contexts (e.g., school), primary health services may overcome some of the barriers against seeking help for mental health problems in youth (Salloum et al., 2016; Reardon et al., 2017).

The relationship between sleep and anxiety is complex, and studies with adults, as well as with children, indicate that associations vary across sleep characteristics (Hudson et al., 2009; van Mill et al., 2014). A population-based study of adolescents reported higher prevalence of daytime sleepiness, SOL, and difficulties getting up in the morning in adolescents with anxiety and in adolescents with depression. However, short sleep duration was only reported by adolescents with depression (Orchard et al., 2020). Thus, to understand in depth the association of specific aspects of sleep with anxiety symptoms in adolescents, we need to include a broad range of sleep characteristics.

Unfortunately, the research literature on sleep and anxiety in youth is characterized by inconsistency regarding what constitutes poor sleep (Leahy and Gradisar, 2012), and the measures used to assess sleep have been questioned (Peterman et al., 2015; Brown et al., 2018). Some studies use a single item to assess insomnia, whereas others measure “sleep-related problems” applying composite scores, with items drawn from anxiety, depression, and/or general child behavioral problem checklists. Items in these composite scores are often related to bedtime behaviors, closely aligned with anxiety (e.g., resistance getting to bed, refusal to sleep alone). This represents a problem of item overlap between sleep and anxiety, that may inflate the sleep-anxiety associations (Peterman et al., 2015). The less frequent use of measures directly assessing sleep duration, SOL and/or insomnia leave us less informed about the nature of the association between sleep and anxiety in adolescents.

Associations between sleep and anxiety in adolescents may vary not only on the aspects of sleep that are measured, but also on the subtypes of anxiety. Some anxiety subtypes may be more strongly associated with sleep difficulties than others. For example, a clinical study reported more sleep-related problems in youth (6–17 years) with generalized anxiety disorder (GAD), and/or separation anxiety disorder (SAD), compared to youth with social anxiety disorder (SoAD) (Alfano et al., 2007). However, two community studies reported high rates of insomnia among adolescents regardless of anxiety subtypes, e.g., across panic disorder, SoAD, GAD, and obsessive-compulsive disorder (OCD) (Johnson et al., 2006a; Alvaro et al., 2017). The inconsistent findings and the limited number of studies suggest that further research on the association between sleep and subtypes of anxiety in adolescents is warranted. This knowledge might be used clinically to determine whether sleep difficulties need to be addressed in youth suffering all forms of anxiety, or only within specific subtypes (e.g., GAD).

Evidence for an association between depression and poor sleep in adolescents is strong (Chorney et al., 2007; Sivertsen et al., 2014; Berger et al., 2019). Higher rates of insomnia have been reported among adolescents with depression, compared to adolescents with anxiety disorders (Johnson et al., 2006a; Alvaro et al., 2017). The high comorbidity between poor sleep and depression, and the large overlap between anxiety and depression, indicate a need to examine how co-occurring depression affects the association between anxiety and sleep. To our knowledge, this has not been explicitly examined in previous studies with adolescents. The role of depression may have implications for the assessment, conceptualization, and interventions offered to adolescents with anxiety.

Adolescent girls usually report higher prevalence of anxiety and depression compared to same age boys (Carter et al., 2011; Leikanger et al., 2012). Sleep has been found to change across development, with a sex specific sleep pattern emerging after puberty, where boys report shorter sleep duration and older adolescents and girls report higher rates of insomnia (Johnson et al., 2006b; Hysing et al., 2013; Amarala et al., 2016). Thus, sex and age need to be considered when examining potential associations between anxiety, depression, and sleep characteristics.

In the present study, sleep (i.e., insomnia, SOL, and sleep duration) was examined in adolescents with anxiety who were seeking help within primary health care. We examined whether total anxiety symptoms and subtypes of anxiety (e.g., GAD and SAD) were associated with sleep duration, SOL, and insomnia. The potential confounding effect of depressive symptoms was also investigated, first by controlling for depressive symptoms in the analyses, and then by examining associations between anxiety and sleep in the subgroup of adolescents without co-occurring depressive symptoms.

## Materials and Methods

Data were drawn from baseline assessments of a randomized controlled trial (RCT) where adolescents with elevated anxiety participated in 10-week, group interventions delivered in junior high schools in Norway. The providers of the group interventions were mainly primary health care workers (i.e., school nurses, *n* = 21; community psychologists, *n* = 5; family therapist, *n* = 1) in addition to professionals from local Child and Adolescent Mental Health Services (*n* = 5, e.g., social workers). Details of the RCT as well as descriptions of interventions, implementation and outcomes have been published elsewhere (Haugland et al., 2017, 2020).

### Procedure and Participants

Participants were informed about the anxiety-focused interventions through multiple sources (e.g., student and parent routine meetings with school nurses; nomination by teachers; information through media). Both self-referral and referral from others were endorsed. Informed consent from caregivers and assent from the adolescents was obtained prior to data collection.

A total of 363 adolescents aged 12–16 years from 18 junior high schools (17 public and 1 private school) were referred for assessment. The schools represented both rural and urban areas. Adolescents were included if the total anxiety score (self-reported or parent-reported anxiety symptoms) was ≥25 on the Spence Children’s Anxiety Scale (SCAS; Spence, 1998). This cut off was slightly above the mean total SCAS score (23.19, *SD* = 15.60) previously found in a community sample of adolescents who attended the same junior high schools as the present sample (Raknes et al., 2017). By applying this cut-off, we included adolescents ranging from mild to more severe levels of anxiety. In addition, a minimum level of interference from anxiety in daily life was required, defined as a score of >1 on the first question on the Child Anxiety Life Interference Scale (CALIS; Lyneham et al., 2013). Exclusion criteria were adolescents having (a) problems following group-rules, (b) disruptive behavior, and/or (c) learning problems causing difficulties following a manualized group-program. Inclusion and exclusion criteria were assessed by providers of the interventions, based on information from adolescents, parents, and teachers. Three adolescents fulfilled exclusion criteria, 34 did not meet inclusion criteria, 7 declined to participate, and six were not included, as some schools did not manage to recruit enough adolescents to form a group before the semester ended. Thus, the final sample comprised 313 adolescents (mean age 14.0 years, *SD* = 0.84, 84.0% girls).

The study was approved by the Regional Committees for Medical and Health Research Ethics (no 2013/2331).

### Instruments

All measures were administered through an online platform. In the present study only self-report data from the adolescents were included. The sleep measures have previously been used in Norwegian population-based studies (Hysing et al., 2013, 2016; Sivertsen et al., 2014, 2017).

#### Demographic Information

All participants indicated their sex, age, and country of birth for themselves and their caregivers. Social class was determined by the highest-ranking occupation between the parents (reported by parents and adolescent), and in accordance with the Registrar General Social Class coding scheme, and categorized as high, medium, and low (Krølner and Holstein, 2006). Family structure was rated from the question “with whom do you live,” with six possible response alternatives, later categorized as either a two-parent or a single parent family.

#### Sleep Characteristics

*Insomnia* was operationalized according to DSM-5 criteria (Hysing et al., 2013). Insomnia comprised a positive response to *Difficulties initiating and maintaining Sleep* (DIMS) with a frequency of at least 3 days per weeks over a period of at least 3 months and a positive response to sleepiness and/or tiredness. DIMS was rated on a three-point Likert-scale with response options “not true,” “somewhat true,” and “certainly true.” Given a positive response (“somewhat true” or “certainly true”), the participants were asked how many days per week they experienced difficulties initiating and maintaining sleep, and how long this had been a problem. *Tiredness/sleepiness* was rated by a joint question on a three-point Likert-scale with response options “not true,” “somewhat true,” and “certainly true.” Given a positive response (“somewhat true” or “certainly true”) adolescents reported the number of days per week they experienced sleepiness and tiredness, respectively.

Youth indicated when they usually went to bed at night and usual rise time in the morning. *Time in bed* (TIB) was calculated by subtracting bedtime from rise-time.

*Sleep onset latency* (SOL) i.e., how long it usually took to fall asleep, was reported in hours and minutes, and further categorized in five levels from “less than 15 min” (lowest scores) to “120 min or more” (highest scores).

*Sleep duration* was calculated separately for weekday nights and weekend nights and defined as TIB minus SOL. Sleep duration was further categorized in ten levels from “less than 4 h” (lowest scores) to “12 h or more “(highest scores).

*Sleep efficiency* was calculated separately for weekday nights and weekend nights, as sleep duration divided by TIB and multiplied by 100 (reported as percentage).

#### Anxiety and Depressive Symptoms

*Anxiety symptoms* were assessed by the Spence Children’s Anxiety Scale (SCAS) (Spence, 1998; Nauta et al., 2004) comprising 44 items, including six positive filler items. SCAS has sound psychometric properties (Nauta et al., 2004; Arendt et al., 2014). Good to excellent internal consistency was found in the current study (*α* = 0.91). In addition to a total anxiety score (i.e., a sum score of all 38 anxiety items), the SCAS comprises six subscales of anxiety along the lines of subtypes of anxiety disorders in the DSM-IV: “separation anxiety” (SAD), “social anxiety” (SoAD), “obsessive compulsive disorder” (OCD), “panic/agoraphobia,” “physical injury fears/simple phobia” (SP) and “generalized anxiety” (GAD). Internal consistency of the anxiety subtypes, each comprising five to nine items, was acceptable for all subscales except for SP (<0.6) (SAD: *α* = 0.91; SoAD: *α* = 0.76; OCD: *α* = 0.76; panic/agoraphobia: *α* = 0.81; SP: *α* = 0.55 and GAD: *α* = 0.78). Due to poor alpha, the SP subtype was removed from further analysis.

*Depressive symptoms* were measured by the Short Mood and Feelings Questionnaire (SMFQ) (Angold et al., 1995), a 13-item scale, with good psychometric properties (Sharp et al., 2006; Lundervold et al., 2013). Good to excellent internal consistency was found in the current sample (*α* = 0.91). To divide the sample into depressed and non-depressed, the SMFQ scores were dichotomized to above clinical cut-off (*n* = 163) and below clinical cut-off (*n* = 150), applying a cutoff score of ≥12 for girls and ≥6 for boys, according to criteria suggested by Jarbin et al. (2020).

### Data Analysis

Demographic characteristics, anxiety scores, depressive symptoms, and sleep characteristics are presented with mean, standard deviation (SD), numbers and percentages. Sex differences in anxiety and depressive symptoms and in sleep variables were analyzed by independent t-tests for continuous variables, and chi-square tests for categorical variables.

Relative risk (RR) with 95% confidence interval (CI) for *insomnia* was estimated for total anxiety symptoms, and anxiety subtypes by log-binomial regression. Three different models were provided: Model 1 unadjusted, model 2 including anxiety, age and sex, and model 3 including anxiety, age, sex, and depressive symptoms. The log-binomial regression failed to converge for the models with total anxiety symptoms and the models with anxiety subtype panic/agoraphobia. To overcome this problem Poisson regression with robust variance estimates was applied to estimated RR (Zou, 2004). In addition, equivalent model 1 and model 2 were applied to estimate the RR for insomnia in the subsample of adolescents scoring below clinical cut-off on depression. For these models there were no problems with convergence.

Linear regression analyses were used to explore associations between total anxiety symptoms, anxiety subtypes, and the sleep measures SOL and sleep duration. Corresponding approach as for the log-binomial regression was applied, with model 1 unadjusted, model 2 including anxiety, age and sex, and model 3 including anxiety, age, sex, and depressive symptoms. Equivalent analyses (model 1 and model 2) regarding SOL and sleep duration were performed within the subsample of adolescents scoring below clinical cut-off on depression. Beta values, 95% CI, standardized beta, and adjusted R^2^ were calculated. Depressive symptoms correlated with the total anxiety symptom score (*r* = 0.67), potentially representing a risk for multicollinearity. However, as the variance inflation factor (VIF) was less than 2.1 for all independent variables in the linear regression analyses, multicollinearity was not considered a problem.

Missing data were low regarding sleep and clinical characteristics (0.6% of values missing, with highest missing for sleep duration 3.5%). Furthermore, data were determined to be missing completely at random, indicated by a non-significant Little’s MCAR test (*p* = 0.312). Due to the low level of missing data, it was considered acceptable to handle missing values by a listwise deletion procedure.

IBM SPSS Statistics 25 (SPSS Inc., Chicago, IL, United States) was used for all analyses, besides the log-binomial regression, where STATA (15.1) (StataCorp, College Station, TX, United States) was used.

## Results

### Demographic and Clinical Characteristics

Mean age of the adolescents was 14.0 years (*SD* = 0.84, 12–16). The sex distribution in the sample was uneven, with 84.0% girls and 16.0% boys (Table 1). Participants were predominately Norwegian (92.0%) (i.e., one or both parents born in Norway), with the majority living in two-parent families (78.8%), and the social class of most families (62.9%) categorized as medium. Table 1 summarizes means and standard deviations for total anxiety symptoms, anxiety symptom subtypes, and depressive symptoms in the study sample.

**TABLE 1:** Baseline demographic information, anxiety and depressive symptoms, and sleep characteristics among adolescents with anxiety (*N* = 313).

		***n***	**%**	***M***	***SD***
**Demographic variables**					
Age				13.99	0.84
Gender:	Female	263	84.0		
Nationality^a^	Norwegian	301	96.2		
Family structure	Two-parent families	246	78.8		
	Single-parent families	66	21.2		
Social class^b^	High	83	26.6		
	Middle	197	62.9		
	Low	32	10.3		
**Internalizing symptoms**					
Total anxiety symptoms (SCAS)				43.44	16.46
SAD				4.77	2.79
SoAD				10.09	3.76
GAD				9.22	3.49
Panic/agoraphobia				7.51	5.48
OCD				6.68	3.69
Depressive symptoms (SMFQ)				11.48	6.81
Below clinical cut-off^c^		150	47.9		
**Sleep characteristics**					
Insomnia (DSM-V)	Yes	119	38.1		
DIMS	Yes	207	66.3		
Tired/sleepy	Yes	283	90.4		
SOL	<30 min	53	16.9		
	30–59 min	58	18.5		
	60–119 min	93	29.7		
	120+ min	109	34.8		
Sleep duration-weekdays	<5 h	36	11.9		
	5–7 h	69	22.9		
	≥7 h	198	65.2		
Sleep efficiency-weekdays	<75	78	25.3		
	75.0–84.9	59	19.2		
	85.0–89.9	60	19.5		
	≥90	111	36.0		

*^a^Norwegian ethnicity defined as 1 or both parents born in Norway.*

*^*b*^Determined by occupation of the highest-ranking parent, in accordance with the Registrar General Social Class coding scheme and categorized as high, medium, and low.*

*^*c*^Clinical cut-off for depressive symptoms ≥6 for boys and ≥12 for girls according to Jarbin et al. (2020).*

*DIMS, difficulties initiating and/or maintaining sleep; GAD, generalized anxiety disorder; OCD, obsessive compulsive disorder; SAD, separation anxiety disorder; SCAS, spence children’s scale; SMFQ, short mood and feeling questionnaire; SOL, sleep onset latency; SoAD, social anxiety disorder*.

Sex differences were found regarding internalizing symptoms, with boys reporting lower total anxiety symptoms and depressive symptoms compared to girls (*p* < 0.001).

### Sleep Characteristics

Of the total sample, 119 adolescents (38.1%) met the criteria for insomnia (Table 1). Significantly more girls (41.2%) compared to boys (22.0%) reported insomnia (*p* = 0.010).

Two thirds of the sample (66.1%) reported having difficulties initiating and maintaining sleep, with significantly more girls (69.5%) than boys (50.0%) (*p* = 0.008). No sex differences were found for the remaining sleep variables.

A large majority of the adolescents (90.4%) reported being tired and/or sleepy during the daytime. A majority (83.1%) reported SOL of 30 min or more, whereas SOL was reported to last for 60 min or more for 64.5%. Sleep duration on weekday nights was below 7 h for 34.8% of the adolescents. Sleep efficiency on weekday nights was below 85.0% for 44.5% of the adolescents.

### Anxiety, Depressive Symptoms, and Sleep

#### Insomnia

Total anxiety symptoms were positively associated with insomnia (RR: 1.03, [95% CI 1.02–1.03], *p* < 0.001) and remained associated with insomnia also after adjusting for age and sex (RR: 1.03, [1.02–1.03], *p* < 0.001) (Table 2). When including depressive symptoms in the model, both total anxiety symptoms (RR: 1.01, [1.01–1.02], *p* = 0.002), and depressive symptoms (RR: 1.04, [1.02–1.07], *p* = 0.001) were associated with insomnia.

**TABLE 2:** Relative risk (RR) of insomnia in adolescents with anxiety by total anxiety symptoms (SCAS), and anxiety subtypes, adjusted for age, sex, and depressive symptoms (SMFQ), including sub-analyses on adolescents below clinical cut-off for depression, estimated from log-binomial regressions.

		**Full sample (*N* = 313)**		**Depression below clinical cut-off^b^ (*n* = 150)**	
		**Insomnia**		**Insomnia**	
	**Independent variables**	**RR**	**95% Cl**	**RR**	**95% Cl**
Model 1	Total anxiety symptoms^a^	1.03***	1.02, 1.03	1.03***	1.02, 1.04
Model 2	Total anxiety symptoms^a^	1.03***	1.02, 1.03	1.03***	1.01, 1.04
Model 3	Total anxiety symptoms^a^	1.01**	1,01, 1.02		
	Depressive symptoms	1.04**	1.02, 1.07		
Model 1	GAD symptoms	1.12***	1.09, 1.14	1.22***	1.11, 1.34
Model 2	GAD symptoms	1.11***	1.07, 1.14	1.20***	1.08, 1.33
Model 3	GAD symptoms	1.04	1.00 1.09		
	Depressive symptoms	1.04*	1.01 1.07		
Model 1	SoAD symptoms	1.10***	1.06, 1.14	1.06	0.99, 1.14
Model 2	SoAD symptoms	1.08***	1.04, 1.12	1.04	0.96, 1.12
Model 3	SoAD symptoms	1.02	0.97, 1.06		
	Depressive symptoms	1.06***	1.03, 1.08		
Model 1	SAD symptoms	1.07***	1.03, 1.11	1.06	0.97, 1.16
Model 2	SAD symptoms	1.06**	1.02, 1.11	1.08	0.98, 1.18
Model 3	SAD symptoms	1.01	0.97, 1.05		
	Depressive symptoms	1.06***	1.04, 1.09		
Model 1	Panic/agoraphobia symptoms^a^	1.08***	1.06, 1.10	1.07***	1.03, 1.12
Model 2	Panic/agoraphobia symptoms^a^	1.07***	1.05, 1.09	1.06*	1.01, 1.10
Model 3	Panic/agoraphobia symptoms^a^	1.04**	1.02, 1.07		
	Depressive symptoms	1.04**	1.02, 1.07		
Model 1	OCD symptoms	1.07***	1.05, 1.10	1.09***	1.04, 1.14
Model 2	OCD symptoms	1.06***	1.04, 1.09	1.10***	1.05, 1.16
Model 3	OCD symptoms	1.01	0.98, 1.04		
	Depressive symptoms	1.06***	1.03, 1.09		

*Model 1: unadjusted; Model 2: adjusted for age and sex; Model 3: including anxiety, age, sex, and depressive symptoms.*

*^*a*^For the full sample Poisson regression was used to estimate RR.*

*^*b*^Clinical cut-off for depressive symptoms ≥6 for boys and ≥12 for girls according to Jarbin et al. (2020).*

GAD, generalized anxiety disorder; OCD, obsessive compulsive disorder; SAD, separation anxiety disorder; SCAS, spence children’s anxiety scale; SMFQ, short mood and feelings questionnaire; SoAD, social anxiety disorder. * p < 0.05, ** p < 0.01, *** p < 0.001.

All anxiety symptom subtypes (i.e., GAD, SoAD, SAD, panic/agoraphobia, and OCD) were associated with insomnia. This was the case also after adjusting for age and sex. When additionally including depressive symptoms in model 3, panic/agoraphobia symptoms remained significantly associated with insomnia, whereas associations between the other anxiety symptom subtypes and insomnia were no longer significant. However, for total anxiety and all anxiety subtypes, when including depressive symptoms in the model, depressive symptoms were associated with insomnia (RR ranging from 1.04 to 1.06, *p* < 0.05).

For the subsample of adolescents scoring below clinical cut-off on depression (*n* = 150) there was an association between anxiety and insomnia (unadjusted RR: 1.03, [1.02–1.04], *p* < 0.001). For the anxiety symptom subtypes, there were associations between insomnia and the anxiety subtypes GAD, panic/agoraphobia, and OCD (Table 2).

#### Sleep Onset Latency

Results from the linear regression analyses showed that total anxiety symptoms were positively associated with SOL (β = 0.25, *p* < 0.001), and remained associated with SOL (β = 0.23, *p* < 0.001) after adjusting for age and sex (Table 3). When including depressive symptoms into the model, total anxiety was no longer associated with SOL, whereas depressive symptoms were (β = 0.28, *p* < 0.001). The full model including total anxiety symptoms, age, sex, and depressive symptoms accounted for 9% of the variance in SOL [*F*(1,310) = 9.02, *p* < 0.001]. All anxiety symptom subtypes were associated with SOL, both before and after adjusting for age and sex (Table 3). However, when including depressive symptoms in the model, the different anxiety subtypes were no longer associated with SOL, whereas depressive symptoms were (β ranging from 0.25 to 0.33, *p* < 0.001).

**TABLE 3:** Associations between sleep onset latency (SOL), total anxiety symptoms (SCAS), and anxiety subtypes, in adolescents with anxiety, adjusting for age, sex, and depressive symptoms (SMFQ), including sub-analyses for youth below clinical cut-off for depression, estimated from linear regression.

		**Full sample (*N* = 313)**				**Depression below clinical cut-off^a^ (*n* = 150)**			
		**Sleep onset latency (SOL)**				**Sleep onset latency (SOL)**			
	**Independent variables**	**β**	***B***	**95% Cl**	**Adj. *R*^2^**	**β**	***B***	**95% Cl**	**Adj. *R*^2^**
Model 1	Total anxiety symptoms	0.25***	0.02	0.01, 0.03	0.06	0.29***	0.03	0.01, 0.05	0.08
Model 2	Total anxiety symptoms	0.23***	0.02	0.01, 0.03	0.06	0.28**	0.03	0.10, 0.05	0.08
Model 3	Total anxiety symptoms	0.06	0.00	−0.01, 0.02	0.09				
	Depressive symptoms	0.28***	0.05	0.02, 0.08					
Model 1	GAD symptoms	0.16**	0.06	0.02, 0.09	0.02	0.12	0.06	−0.02, 0.13	0.01
Model 2	GAD symptoms	0.14*	0.05	0.01, 0.09	0.03	0.11	0.05	−0.02, 0.13	0.02
Model 3	GAD symptoms	−0.04	−0.01	−0.06, 0.03	0.09				
	Depressive symptoms	0.33***	0.06	0.03, 0.08					
Model 1	SoAD symptoms	0.20***	0.07	0.03, 0.10	0.04	0.20*	0.07	0.02, 0.13	0.04
Model 2	SoAD symptoms	0.18**	0.06	0.02, 0.10	0.04	0.18*	0.06	0.00, 0.12	0.04
Model 3	SoAD symptoms	0.01	0.00	−0.04, 0.05	0.09				
	Depressive symptoms	0.30***	0.05	0.03, 0.08					
Model 1	SAD symptoms	0.12*	0.05	0.00, 0.10	0.01	0.14	0.07	−0.01, 0.15	0.01
Model 2	SAD symptoms	0.12*	0.05	0.00, 0.10	0.02	0.15	0.08	0.00, 0.15	0.03
Model 3	SAD symptoms	0.01	0.00	−0.05, 0.05	0.09				
	Depressive symptoms	0.31***	0.06	0.03, 0.08					
Model 1	Panic/agoraphobia symptoms	0.26***	0.06	0.03, 0.08	0.06	0.25**	0.08	0.03, 0.14	0.06
Model 2	Panic/agoraphobia symptoms	0.24***	0.05	0.03, 0.08	0.06	0.23**	0.08	0.02, 0.13	0.06
Model 3	Panic/agoraphobia symptoms	0.09	0.02	−0.01, 0.05	0.10				
	Depressive symptoms	0.25**	0.04	0.02, 0.07					
Model 1	OCD symptoms	0.20***	0.07	0.03, 0.10	0.04	0.24**	0.09	0.03, 0.15	0.05
Model 2	OCD symptoms	0.19**	0.06	0.03, 0.10	0.04	0.25**	0.09	0.03, 0.15	0.07
Model 3	OCD symptoms	0.07	0.02	−0.02, 0.06	0.09				
	Depressive symptoms	0.27***	0.05	0.03, 0.07					

*Model 1: unadjusted, Model 2: adjusted for age and sex, Model 3: including anxiety, age, sex, and depressive symptoms,*

*^*a*^clinical cutoff ≥12 for girls and ≥6 for boys according to Jarbin et al. (2020).*

β, standardized beta; GAD, generalized anxiety disorder; OCD, obsessive compulsive disorder; SAD, separation anxiety disorder; SCAS, spence children’s anxiety scale; SMFQ, short mood and feelings questionnaire; SoAD, social anxiety disorder; *p < 0.05, **p < 0.01, ***p < 0.001.

For the subsample of adolescents below clinical cut-off on depressive symptoms (*n* = 150), an association was found between total anxiety symptoms and SOL (β = 0.29, *p* ≤ 0.001), as well as associations between symptoms of SoAD, Panic/agoraphobia, OCD and SOL (Table 3).

#### Sleep Duration

Total anxiety symptoms were negatively associated with sleep duration (β = −0.18, *p* < 0.01), and remained associated with sleep duration (β = −0.15, *p* < 0.01) after adjusting for age and sex (Table 4). When including depressive symptoms in the model, total anxiety was no longer associated with sleep duration, whereas depressive symptoms were (β = −0.30, *p* < 0.001). The model including total anxiety symptoms, age, sex, and depressive symptoms accounted for 8% of the variance in sleep duration [*F*(1,299) = 7.60, *p* < 0.001].

**TABLE 4:** Associations between sleep duration, total anxiety symptoms (SCAS), and anxiety subtypes in adolescents with anxiety, adjusting for age, sex, and depressive symptoms (SMFQ), including a subanalyses for adolescents below clinical cut-off on depression, estimated from linear regression.

		**Full sample (*N* = 313)**				**Depression below clinical cut-off^a^ (*n* = 150)**			
		**Sleep duration wk**				**Sleep duration wk**			
	**Independent variables**	**β**	***B***	**95% Cl**	**Adj. *R*^2^**	**β**	***B***	**95% Cl**	**Adj. *R*^2^**
Model 1	Total anxiety symptoms	−0.18**	−0.02	−0.03, −0.01	0.03	−0.15	−0.02	0.04, 0.00	0.02
Model 2	Total anxiety symptoms	−0.15**	−0.02	−0.03, 0.00	0.04	−0.16	−0.02	−0.04, 0.00	0.05
Model 3	Total anxiety symptoms	0.03	0.00	−0.01, 0.02	0.08				
	Depressive symptoms	−0.30***	−0.07	−0.11, −0.04					
Model 1	GAD symptoms	−0.10	−0.05	−0.11, 0.00	0.01	−0.02	−0.01	−0.11, 0.08	−0.01
Model 2	GAD symptoms	−0.08	−0.04	−0.10, 0.02	0.02	−0.03	−0.01	−0.10, 0.08	0.03
Model 3	GAD symptoms	0.10	0.05	−0.02, 0.11	0.09				
	Depressive symptoms	−0.33***	−0.08	−0.12, −0.05					
Model 1	SoAD symptoms	−0.17**	−0.08	−0.13, −0.03	0.03	−0.07	−0.03	−0.10, 0.04	0.00
Model 2	SoAD symptoms	−0.14*	−0.06	−0.12, −0.01	0.03	−0.06	−0.03	−0.10, 0.05	0.04
Model 3	SoAD symptoms	0.02	0.01	−0.05, 0.07	0.08				
	Depressive symptoms	−0.28***	−0.07	−0.11, −0.04					
Model 1	SAD symptoms	−0.01	0.00	−0.07, 0.07	0.00	0.01	0.01	−0.09, 0.10	−0.01
Model 2	SAD symptoms	−0.01	0.00	−0.07, 0.06	0.01	−0.01	0.00	−0.10, 0.09	0.03
Model 3	SAD symptoms	0.11	0.07	−0.01, 0.14	0.09				
	Depressive symptoms	0.32***	−0.08	−0.11, −0.05					
Model 1	Panic/agoraphobia symptoms	−0.21***	−0.07	−0.01, −0.03	0.04	−0.18*	−0.07	−0.14, −0.01	0.02
Model 2	Panic/agoraphobia symptoms	−0.18**	−0.06	−0.09, −0.02	0.05	−0.16	−0.07	−0.13, 0.00	0.06
Model 3	Panic/agoraphobia symptoms	−0.04	−0.01	−0.06, 0.03	0.08				
	Depressive symptoms	−0.25**	−0.06	−0.10, −0.03					
Model 1	OCD symptoms	−0.13*	−0.06	−0.11, −0.01	0.01	−0.13	−0.06	−0.13, 0.01	0.01
Model 2	OCD symptoms	−0.12*	−0.06	−0.11, 0.00	0.03	−0.16	−0.07	−0.14, 0.00	0.06
Model 3	OCD symptoms	0.00	0.00	−0.06, 0.06	0.08				
	Depressive symptoms	−0.27***	−0.07	−0.10, −0.04					

*Model 1: unadjusted, Model 2: adjusted for age and sex, Model 3: adjusted for age, sex, and depressive symptoms.*

*^*a*^Clinical cutoff ≥12 for girls and ≥6 for boys according to Jarbin et al. (2020).*

β, standardized beta; GAD, generalized anxiety disorder; OCD, obsessive compulsive disorder; SAD, separation anxiety disorder; SCAS, spence children’s anxiety scale; SMFQ, short mood and feelings questionnaire; SoAD, social anxiety disorder.

*p < 0.05, **p < 0.01, ***p < 0.001.

In the unadjusted models, the subtypes SoAD, Panic/agoraphobia, and OCD were associated with sleep duration, with higher levels of anxiety associated with lower sleep duration. These anxiety subtypes remained associated with sleep duration after adjusting for age and sex. However, when entering depressive symptoms to the model, none of the anxiety subtypes were associated with sleep duration, whereas associations between depressive symptoms and sleep duration emerged (β ranging from −0.25 to −0.33, *p* < 0.001).

For the subsample of adolescents below clinical cut-off on depressive symptoms (*n* = 150), no association was found between total anxiety symptoms and sleep duration. There were no associations between the subtypes of anxiety and sleep duration, except for the unadjusted association between symptoms of panic/agoraphobia and sleep duration (Table 4).

## Discussion

The findings indicate that sleep should be addressed among adolescents with anxiety seeking help from primary health care services. A considerable proportion of the adolescents (12–16 years) in this study reported high rates of insomnia, long sleep onset latency (SOL), and short sleep duration. Self-reported anxiety was positively associated with insomnia and SOL across all subtypes of anxiety, indicating that we need to address insomnia and SOL in adolescents regardless of anxiety subtypes. Also, some of the anxiety subtypes (i.e., SoAD, panic/agoraphobia, and OCD) were associated with sleep duration. Although co-occurring depressive symptoms accounted for most of the associations between anxiety and the sleep variables, associations between anxiety and insomnia, as well as SOL, were still found among adolescents who scored below clinical cut-off on depressive symptoms. Hence, poor sleep should be addressed among adolescents with elevated anxiety, even in the absence of co-occurring depression.

### Sleep Duration, Sleep Onset Latency, and Insomnia in Adolescents With Anxiety

The 38.1% insomnia rate in the present sample was considerably higher than a prevalence of 18.5% found in a general population-based study of Norwegian adolescents, using the same definition of insomnia (Hysing et al., 2013). Also, the percentage of adolescents reporting SOL ≥30 min, was higher in the sample of adolescents with anxiety (83.1%) compared to the adolescents in the population-based study (65%) (Hysing et al., 2013). However, as the population-based sample comprised older adolescents (16–18 years) compared to the current sample of adolescents with anxiety (12–16 years), the comparisons must be interpreted with caution. Other results from the same population-based study, however, indicate that sleep problems increase during late adolescence (Sivertsen et al., 2017). Thus, based on age alone, adolescents in the current sample should have had better, and not poorer sleep, compared to the population-based sample.

In line with previous findings of increased SOL in youth with anxiety disorders (Forbes et al., 2008; Orchard et al., 2020), most of the participants in the current study reported long SOL, with over half of the adolescents (64.5%) spending more than 1 h each night trying to fall asleep. The prevalence of insomnia in the present sample (38.1%) also appears to be higher than the 10.7% rate of insomnia found in a population-based study of American adolescents (13–16 years), using similar methodology to measure insomnia (Johnson et al., 2006a). The rate of insomnia in our sample was more in line with, and even somewhat higher than, the rate of insomnia (25.6%) reported by adolescents who fulfilled criteria for anxiety disorders in the same study (Johnson et al., 2006a).

About one third (35%) of the adolescents slept less than 7 h on weekday nights, which is considered short, according to expert-based guidelines recommending 8–10 h of sleep for teenagers (Hirshkowitz et al., 2015). However, an even higher percentage in the Norwegian population-based sample (53.8%) reported sleep duration below 7 h on weekday nights (Hysing et al., 2016). This may be explained by a general decrease in hours of sleep occurring during the later adolescent years (Carskadon, 2011), given that the population-based sample had a somewhat higher age range than our sample. However, a previous study reported no difference between adolescents with and without anxiety disorders regarding sleep duration (Orchard et al., 2020). Thus, it is possible that sleep duration might be less affected by anxiety, and that adolescents with anxiety have no higher risk of short sleep duration than their peers. However, the limited evidence available, together with the high rates of insomnia and long SOL in the present sample, suggests that we need further studies before drawing firm conclusions regarding the association between anxiety and sleep duration.

In the present study, more girls met criteria for insomnia compared to boys. This is in accordance with sex differences found in studies of adolescents in the general population (Johnson et al., 2006b; Hysing et al., 2013). In the current sample, no difference was found between boys and girls regarding sleep duration, whereas shorter sleep duration was found among boys compared to girls in the comparison population-based study (Hysing et al., 2013). However, given the limited number of boys included in the present sample, the lack of sex differences should be interpreted with caution. In general, adjusting for sex and age did not change the associations between anxiety and sleep variables in this study. Total anxiety symptoms and most anxiety subtypes were associated with insomnia, SOL, and sleep duration even after sex and age were statistically controlled.

### Anxiety Subtypes and Sleep

All anxiety subtypes were associated with insomnia and SOL, whereas sleep duration was negatively associated with symptoms of SoAD, panic/agoraphobia, and OCD. Previous research on associations between sleep and subtypes of anxiety is limited.

In this study subscales on the SCAS, and not diagnostic categories, were used to assess anxiety subtypes. However, a study comparing SCAS subscales with scores from diagnostic interviews, has shown the SCAS subscales to validly assess SoAD, GAD, panic/agoraphobia, OCD, and SAD in adolescents (Olofsdotter et al., 2015).

While we have not specifically addressed the factors that might drive the high prevalence of insomnia and long SOL, previous research with youth with anxiety disorders suggests that this may partly be explained by heightened physical and cognitive arousal, poorer emotion regulation, and vigilance to threat, all factors that may make sleep harder to initiate in youth with anxiety (Palmer and Alfano, 2017; Blake et al., 2018; Ricketts et al., 2018). These factors are probably common features of anxiety, explaining why sleep problems may be found across different anxiety subtypes.

### The Role of Co-occurring Depressive Symptoms

With a few exceptions for insomnia, when depressive symptoms were included in the regression models, anxiety was no longer associated with the sleep variables. Strong associations between depression and sleep have been explained by excessive worrying, where worrying seems to precede and partly mediate the association between poor sleep and depressive symptoms in adolescents (Danielsson et al., 2013). Poor sleep may furthermore be related to poorer mood regulation, indicating a potential mechanism from poor sleep to mood disorders in adolescents (Short et al., 2020).

An alternative interpretation could be that the strong association found between depressive symptoms and sleep is a result of informant-bias – in other words, depressed youth may generally report more negative experiences due to their negative perceptions of the world. Support for this possibility is found in a study of youth (7–17 years) where those with anxiety disorders showed more sleep problems on objective sleep measures compared to depressed and non-clinical youth, whereas according to self-report, youth with anxiety had fewer sleep problems than those with depression (Forbes et al., 2008).

To further explore the association between anxiety, depressive symptoms, and sleep, we examined a subsample of adolescents scoring below clinical cutoff on depressive symptoms. In this subsample, anxiety symptoms remained associated with insomnia and SOL. Thus, anxiety and sleep difficulties were associated also among adolescents with anxiety who reported low levels of depression. Common factors have been suggested to underly the associations between anxiety, depression, and sleep (Blake et al., 2018). Adolescents with sleep disturbances have been found to worry more about issues such as bullying, terrorist attacks, illness, and accidents, compared to adolescents who sleep well (Danielsson et al., 2016). These worries are also frequently found among youth with high levels of anxiety (Weems et al., 2000).

Perseverative cognition is repetitive cognitive processes constituting the common features between worrying and rumination. These cognitions have been found to prolong stress-related affective and physiological activation, in advance of and following stressors (Brosschot et al., 2006). The prolonged activation can make initiating and maintaining sleep difficult, and perseverative cognitions could therefore explain the long SOL and the high prevalence of insomnia found among adolescents with anxiety in the current study. Perseverative cognitions are commonly associated with both anxiety and depression (Brosschot et al., 2006), and might be a possible underlying mechanism in the association between depressive and anxiety symptoms, and sleep problems.

### Strengths and Limitations

The current study expands on previous knowledge by examining sleep in a sample of adolescents with anxiety referred to early intervention. Examining sleep variables in this sample may increase our knowledge of problems and needs among adolescents seeking help within primary health care services.

A further strength of the study was the inclusion of measures specifically assessing insomnia, SOL, and sleep duration, rather than composite sleep measures that include overlapping items between sleep and anxiety. The focus on adolescents’ self-reports rather than parent-reports is also a strength given that many sleep difficulties (e.g., long SOL) and anxiety symptoms are not always easily observable by caregivers. Finally, exploring the role of depressive symptoms in the associations between anxiety and sleep is novel, and needs to be explored further.

The study has several limitations. First, the cross-sectional design precludes any conclusions about causality or directionality between anxiety symptoms, depressive symptoms, and sleep. Second, no comparison group was included, making conclusions about level of sleep problems less definitive.

The large difference in the number of boys versus girls limits interpretations about sex differences. Whereas the exclusive use of subjective measures of sleep is a limitation, a recent study reports high correspondence between subjective and objective measures of sleep among adolescents (Lucas-Thompson et al., 2020). Furthermore, sleep duration was based on SOL subtracted from time in bed. To get a more accurate measure, wake after sleep onset should also have been subtracted from time in bed. Wake after sleep onset was not assessed but is generally quite short during adolescence. However, this could have led to an overestimation of sleep duration in the present study. For insomnia, a clinical interview is considered the gold standard, and this would have strengthened the validity of the insomnia category.

## Conclusion

Adolescence is a period characterized by increase in sleep problems and, at the same time, anxiety and depressive symptoms commonly increase. The co-occurrence of these problems may cause considerable impairment during these critical years of a young person’s development. The high rate of insomnia and the long SOL found among adolescents with different subtypes of anxiety, suggest a need to assess sleep among all adolescents who seek help for anxiety within primary health care services, regardless of subtype of anxiety they present with. The findings further point to the need to concurrently assess depressive symptoms, which may account for a considerable portion of the common variance between anxiety and the sleep variables. Nonetheless, given that anxiety is related to insomnia and SOL even among anxious adolescents who report low levels of depression, it is important to assess sleep in all adolescents presenting with anxiety.

Interventions need to address the complex symptomatology often presented by adolescents with elevated anxiety. Cognitive behavioral therapy (CBT) programs frequently used for treatment or indicated prevention for adolescents with anxiety do not focus on potential co-occurring sleep problems (e.g., Coping Cat, Kendall and Hedtke, 2006; Cool Kids, Rapee et al., 2006). This makes it highly possible that sleep problems in adolescents with anxiety are neither recognized, nor treated. Nonetheless, a few approaches targeting sleep in adolescents, either across mental health problems (e.g., Harvey, 2016), or specifically aiming to reduce co-occurring sleep problems and anxiety (Blake et al., 2016) have been developed and evaluated.

Evidence suggests that CBT for insomnia may reduce anxiety (Belleville et al., 2011), perhaps through reducing anxious worrying (Ballesio et al., 2021). Studies have also examined the integration of CBT for insomnia with techniques specifically targeting anxious worrying (Ballesio et al., 2021). As this is research including only adult samples, further studies need to examine if CBT for insomnia have effect on anxious worrying also in adolescent samples.

In general, approaches integrating interventions for anxiety and sleep problems specifically targeting adolescents need to be developed, evaluated, and disseminated in primary health care settings.

## Data Availability Statement

The datasets generated for this study are not readily available because confidential patient data are included. All requests must be approved by the Norwegian Regional Committees for Medical and Health Research Ethics. Requests to access the datasets should be directed to BH, bente.haugland@uib.no.

## Ethics Statement

The study involved human participants and was reviewed and approved by the Regional Committee for Medical and Health Research Ethics in Western Norway (no. 2013/2331). Written informed consent to participate in this study was provided by the participants’ legal guardian/next of kin.

## Author Contributions

All authors contributed to the study conception and design. BH, MH, VB, GW, RR, AH, TH, and JB performed the material preparation, data collection, and analysis. BH wrote the first draft of the manuscript. All authors commented on the different versions of the manuscript and read and approved the final manuscript.

## Conflict of Interest

The authors declare that the research was conducted in the absence of any commercial or financial relationships that could be construed as a potential conflict of interest.
